# Essential role of caspase-8 in p53/p73-dependent apoptosis induced by etoposide in head and neck carcinoma cells

**DOI:** 10.1186/1476-4598-10-95

**Published:** 2011-07-31

**Authors:** Juan Liu, Hiroshi Uematsu, Nobuo Tsuchida, Masa-Aki Ikeda

**Affiliations:** 1 Section of Molecular Embryology, Graduate School of Medical and Dental Sciences, Tokyo Medical and Dental University, Tokyo, Japan; 2 Section of Molecular Cellular Oncology and Microbiology, Graduate School of Medical and Dental Sciences, Tokyo Medical and Dental University, Tokyo, Japan; 3 Section of Gerodontology, Graduate School of Medical and Dental Sciences, Tokyo Medical and Dental University, Tokyo, Japan

## Abstract

**Background:**

Caspase-8 is a key upstream mediator in death receptor-mediated apoptosis and also participates in mitochondria-mediated apoptosis via cleavage of proapoptotic Bid. However, the role of caspase-8 in p53- and p73-dependent apoptosis induced by genotoxic drugs remains unclear. We recently reported that the reconstitution of procaspase-8 is sufficient for sensitizing cisplatin- but not etoposide-induced apoptosis, in chemoresistant and caspase-8 deficient HOC313 head and neck squamous cell carcinoma (HNSCC) cells.

**Results:**

We show that p53/p73-dependent caspase-8 activation is required for sensitizing etoposide-induced apoptosis by utilizing HOC313 cells carrying a temperature-sensitive p53G285K mutant. Restoration of wild-type p53 function under the permissive conditions, together with etoposide treatment, led to substantial transcriptional activation of proapoptotic Noxa and PUMA, but failed to induce apoptosis. In addition to p53 restoration, caspase-8 reconstitution was needed for sensitization to etoposide-induced apoptosis, mitochondria depolarization, and cleavage of the procaspases-3, and -9. In etoposide-sensitive Ca9-22 cells carrying a temperature-insensitive mutant p53, siRNA-based p73 knockdown blocked etoposide-induced apoptosis and procaspase-8 cleavage. However, induction of p73 protein and up-regulation of Noxa and PUMA, although observed in Ca9-22 cells, were hardly detected in etoposide-treated HOC313 cells under non-permissive conditions, suggesting a contribution of p73 reduction to etoposide resistance in HOC313 cells. Finally, the caspase-9 inhibitor Ac-LEHD-CHO or caspase-9 siRNA blocked etoposide-induced caspase-8 activation, Bid cleavage, and apoptosis in both cell lines, indicating that p53/p73-dependent caspase-8 activation lies downstream of mitochondria.

**Conclusions:**

we conclude that p53 and p73 can act as upstream regulators of caspase-8, and that caspase-8 is an essential mediator of the p53/p73-dependent apoptosis induced by etoposide in HNSCC cells. Our data suggest the importance of caspase-8-mediated positive feedback amplification in the p53/p73-dependent apoptosis induced by etoposide in HNSCC cells.

## Background

Apoptosis or programmed cell death plays an essential role in the development and homeostasis of multicellular organisms [1,2]. Because many anticancer drugs kill tumor cells by inducing apoptosis, mutations or dysregulation of pro- and anti-apoptotic proteins can contribute to the acquisition of chemoresistance.

Two major apoptotic pathways have been defined in mammalian cells: the extrinsic death receptor pathway and the intrinsic mitochondrial pathway [3,4]. The extrinsic pathway is initiated by the binding at the plasma membrane of death ligands (e.g. FasL, TNF-α, TRAIL) to their death receptors, which belong to the tumor necrosis factor (TNF) receptor superfamily members (e.g. Fas/Apo1, KILLER/DR5, TNF-RI, TRAIL receptor). In contrast, the intrinsic pathway is initiated by signals from within the cell to induce the apoptotic process via the release of cytochrome *c *and other pro-apoptotic proteins from mitochondria. Apoptosis is executed by a family of cysteine-dependent aspartate-directed proteases (caspases). Based on their function, caspases are classified into two groups, initiator caspases (e.g. caspase-8 and -9) and effector caspases (e.g. caspase-3, -6, and -7). Caspase-8 is predominantly activated by signals from the death receptor pathway, while caspase-9 activation is dependent primarily on the mitochondrial pathway. In both apoptotic pathways, these initiator caspases activate downstream effector caspases (e.g. caspases-3, -6, -7) by a proteolytic cascade, resulting in the cleavage of a variety of cellular substrates involved in apoptosis.

Although the apoptosis induced by genotoxic drugs is generally thought to be dependent on mitochondria-mediated caspase-9 activation, a number of studies have reported caspase-8 activation during drug-induced apoptosis [5-22]. Drug-induced caspase-8 activation has been shown to occur not only via the death receptor pathway, but also via the mitochondrial pathway [11-22]. Caspase-8 can be activated downstream of caspase-9, through caspases-3 and -6, independently of death receptor signalling [17-19,23]. Furthermore, caspase-8 can amplify the death signal by activating the mitochondrial pathway through the cleavage of the BH3-only protein Bid [14,24-30]. Cleaved Bid (tBid) translocates to the mitochondria and then triggers mitochondrial depolarization, leading to cytochrome *c *release and subsequent caspase-9 activation, by which the activation of caspase-8 initiates a positive feedback loop that amplifies the mitochondrial pathway.

The p53 family proteins (p53, p63, and p73) regulate apoptotic pathways upstream of caspases in response to genotoxic drugs through transcriptional activation of proapoptotic genes, the products of which participate in the major apoptotic pathways: TNF receptor superfamily members (Fas/Apo1 and KILLER/DR5) in the death receptor pathway, and pro-apoptotic Bcl-2 family members (Bax, Puma, Noxa and Bid) in the mitochondrial pathway [31-34]. Transcriptionally independent functions of p53 also affect the mitochondrial pathway, as p53 localizes either in the cytosol or at the mitochondria, triggering mitochondrial depolarization via activation of Bax or Bak [35]. Recent studies of the p53 family members p63 and p73 have indicated that p53, p63 and p73 jointly mediate cellular responses to genotoxic drugs [36]. TA-p73 is induced by a variety of drugs and can compensate for deficient p53 function so as to induce apoptosis in p53-deficient tumors [37-39]. Although *p63 *and *p73 *gene mutations are rare, it has been reported that p63 levels and p73 status are important determinants of responsiveness to cytotoxic drugs in HNSCC [38-40]. Nevertheless, the precise mechanisms by which p53 family members regulate caspase activation during drug-induced apoptosis are not presently well understood.

Previously, we examined the functional relevance of caspase-8 in drug-induced apoptosis by using the *p53*-mutated HOC313 HNSCC cell line, which is highly resistant to TRAIL and chemotherapeutic drugs, including cisplatin and etoposide [41,42]. We found a biallelic caspase-8 nonsense mutation that led to the truncation of all of its defined functional domains in HOC313 cells. Reconstitution of caspase-8 by stable transfection of wild-type *procaspase-8 *sensitized the cells to cisplatin-induced apoptosis, in parallel with cisplatin-induced up-regulation of TNF-α and TRAIL mRNA. However, while caspase-8 activation by etoposide treatment has been described in other types of cells [11,19-22], caspase-8 reconstitution was insufficient to sensitize HOC313 cells to etoposide-induced apoptosis in the HOC313 cell line, suggesting additional mechanisms of resistance to etoposide in this cell line.

Given the importance of p53 as an upstream regulator of caspase activation, we hypothesized that p53 and its family members may be involved in caspase-8 activation and the induction of apoptosis by etoposide in HNSCC cells. In this study, we explored the functional relevance of caspase-8 and the p53 status in etoposide-induced apoptosis by using two HNSCC cell lines: an etoposide-resistant HOC313 cell line that is deficient in caspase-8 which carries the temperature-sensitive mutant p53G285K; and etoposide-sensitive Ca9-22 cell line that expresses wild-type caspase-8 and the p53R248W mutant, which has no recognizable temperature-sensitive properties. Our data demonstrate a critical role for p53/p73-dependent caspase-8 activation in etoposide-induced apoptosis in HNSCC cells.

## Results

### The restoration of wild-type p53 function induced p53-target gene expression in etoposide-treated HOC313 cells

**Figure 1:**
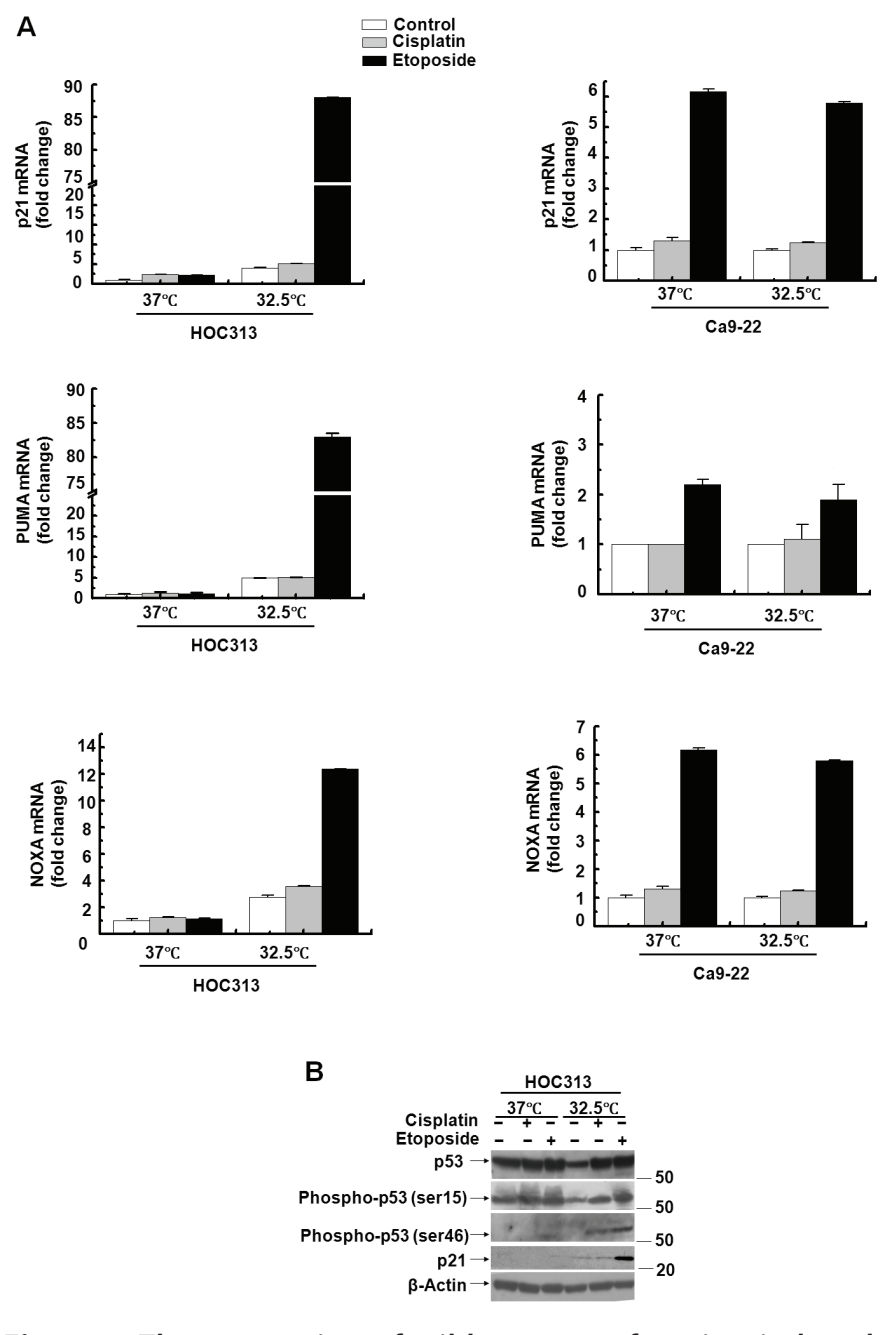
**The restoration of wild-type p53 function induced p53-target gene expression in etoposide-treated HOC313 cells**. A, cells treated with cisplatin (10 μg/ml) or etoposide (100 μg/ml) were incubated at 32.5°C or 37°C for 6 h; the amount of mRNA encoding PUMA, Noxa and p21 was measured using real-time RT-PCR. n > 3 for all conditions. Bars, SD. B, p53 phosphorylation and p21 expression in drug-treated cells. Cells were treated with drugs as in A for 24 hours followed by Western blot analysis with the indicated antibodies. β-actin was used as a loading control.

We previously reported that stable caspase-8 reconstitution sensitizes drug-resistant and *caspase-8*-deficient HOC313 cells to cisplatin-induced apoptosis [42]. However, caspase-8 reconstitution was insufficient to sensitize HOC313 cells to apoptosis induced by etoposide. To explore the mechanisms underlying etoposide resistance in HOC313 cells, we examined the role of p53 function in etoposide-induced apoptosis by temperature-downshift experiments, as HOC313 cells express the endogenous mutant p53G285K protein which has been shown to have temperature-sensitive properties [43]. CAT reporter assays confirmed that transfection of Saos-2 cells with a plasmid expressing p53G285K led to a 32.3-fold increase in the transactivation of a p53 reporter gene containing p53-responsive elements at 32.5°C, while no activation (0.9-fold) was observed at 37°C. Furthermore, quantitative real-time RT-PCR analysis of p53-target genes revealed that a temperature downshift to 32.5°C resulted in a 22-fold increase compared to the untreated control in mRNA expression of the p21^Waf1/Cip1 ^gene, a critical mediator of p53-induced cell-cycle arrest, in etoposide- but not cisplatin-treated HOC313 cells (Figure 1A). This occurred in parallel with induction of p21^Waf1/Cip1 ^protein as demonstrated by Western blotting (Figure 1B). This temperature downshift also led to 17- and 4.5-fold increases of proapoptotic PUMA and Noxa transcripts, respectively, in etoposide- but not cisplatin-treated HOC313 cells, as compared to the untreated controls. In Ca9-22 cells, which express the temperature-insensitive p53R248W mutant, etoposide, but not cisplatin treatment led to up-regulation of p21, PUMA and Noxa transcription equally at both temperatures (Figure 1A).

Numerous studies have demonstrated that phosphorylation of p53 at specific N-terminal serines in response to DNA damage contributes to increase the stability and activity of p53 [31-34]. Among them, ser46 phosphorylation has been reported to enhance p53-mediated transcriptional activation of pro-apoptotic genes [44]. While the temperature downshift alone led to a slight increase of p53-target gene expression in HOC313 cells (Figure 1A), it did not induce Ser46 phosphorylation in the absence of drug treatment (Figure 1B). Etoposide and, to a lesser extent, cisplatin treatment caused ser46 phosphorylation following the temperature downshift in HOC313 cells, while each of the drugs induced ser15 phosphorylation at both temperatures. Notably, ser46 phosphorylation of the p53G285K protein was hardly detected after drug treatment at 37°C in HOC313 cells, which is consistent with previous observations that a certain type of mutant p53 is not susceptible to drug-induced Ser46 phosphorylation [45]. Collectively, these data indicate that the temperature downshift to the permissive temperature (32.5°C) leads to the restoration of mutant p53G285K to the wild-type p53 function, thereby leading to transcriptional induction of p53-target genes in etoposide- but not cisplatin-treated HOC313 cells. In addition, the fact that the expression of p53-target genes was unaffected by cisplatin indicates that these two drugs have distinct effects on p53-target gene expression in HNSCC cell lines.

### Both p53 restoration and caspase-8 reconstitution are needed for sensitizing etoposide-induced apoptosis in HOC313 cells

**Figure 2:**
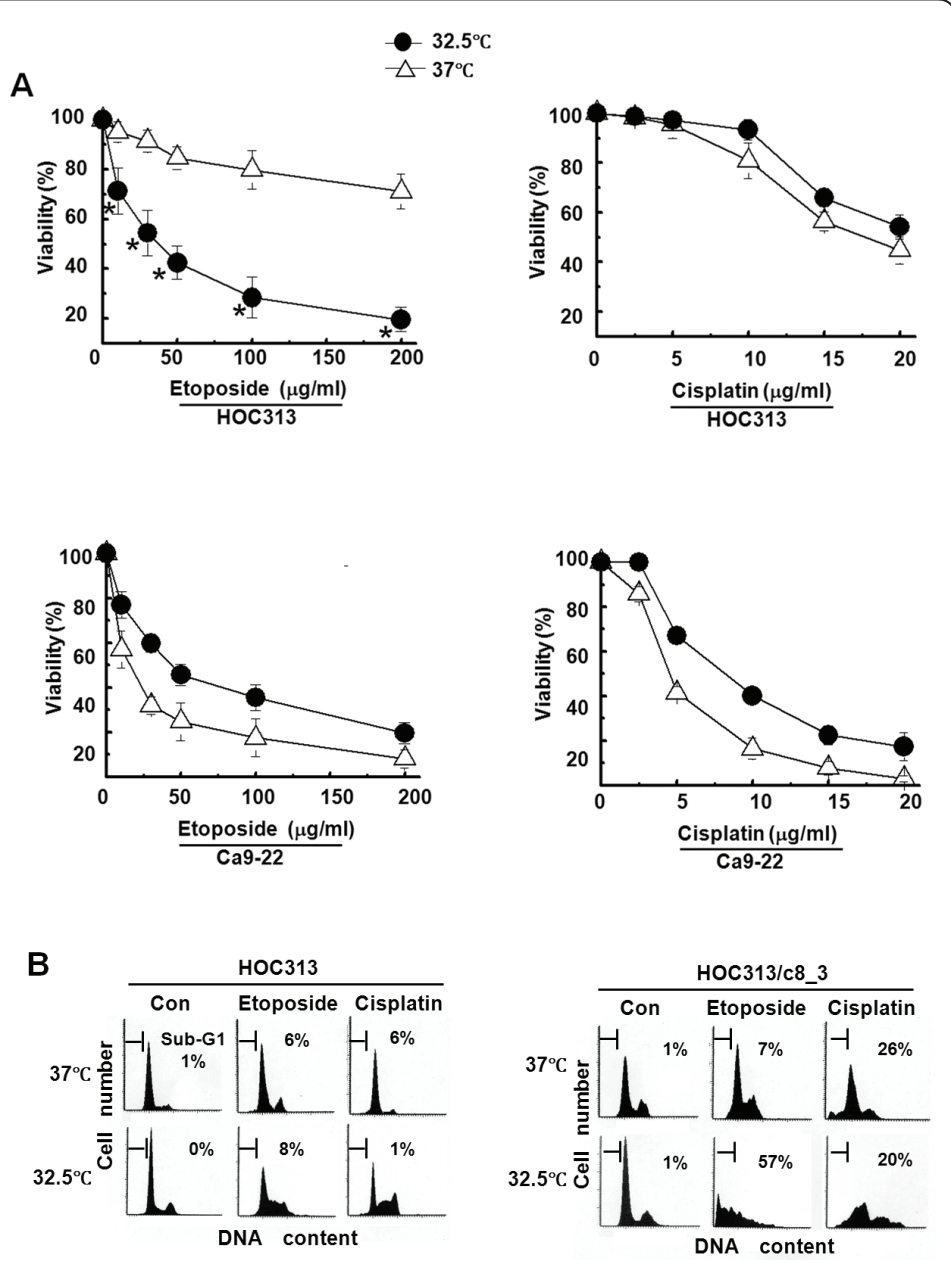
**Both p53 restoration and caspase-8 reconstitution are needed to sensitize etoposide-induced apoptosis in HOC313 cells**. A, effects of temperature downshift on the chemosensitivity of HOC313 and Ca9-22 cells. Cells were treated with increasing doses of cisplatin or etoposide at 32.5°C (solid circle) or 37°C (open triangle) for 24 h. Cell viability was measured by WST-1 assay. The results are the means ± SD from three independent experiments. Bars, SD. The asterisk indicates a significant difference (p < 0.05) between 32.5°C and 37°C (*t*-test). B, effects of temperature downshift on the DNA profile of drug-treated HOC313 and HOC313/c8_3 cells. Cells treated with cisplatin (10 μg/ml) or etoposide (100 μg/ml) were incubated at 32.5°C or 37°C for 24 h, followed by LSC analysis. The horizontal and vertical axes represent the DNA content and cell number, respectively. The apoptotic cell population is indicated as a percentage of the sub-G1 fraction.

We examined whether the restoration of wild-type p53 function affects the chemosensitivity of HOC313 cells by using drug-sensitive Ca9-22 cells as the control. Cell viability assays under permissive and non-permissive conditions revealed that p53 restoration by temperature downshift to 32.5°C significantly sensitized HOC313 cells to etoposide, but not cisplatin (Figure 2A). However, the temperature downshift rendered Ca9-22 cells to become slightly more resistant to both etoposide and cisplatin.

We next investigated the functional role of caspase-8 in etoposide-induced apoptosis by using HOC313/c8_3 cells stably reconstituted for caspase-8 [42]. Laser scanning cytometry (LSC) analysis showed that p53 restoration resulted in no increase of the sub-G_1 _population in either the cisplatin- or etoposide-treated parent HOC313 cells (Figure 2B). By contrast, in HOC313/c8_3 cells, p53 restoration induced apoptosis (sub-G_1 _population = 57%) after etoposide treatment, while cisplatin induced apoptosis without the temperature downshift, as reported previously [42]. In addition, no induction of apoptosis and Ser46 phosphorylation (Figure 1B) by the temperature downshift in the absence of drug treatment indicates that the temperature downshift by itself is insufficient to fully activate the restored p53G285K protein in HOC313 cells. These results demonstrate that functions of both p53 and caspase-8 are required for the sensitization to etoposide-induced apoptosis in HOC313 cells.

### Both p53 and caspase-8 functions are needed for etoposide-induced caspase activation in HOC313 cells

**Figure 3:**
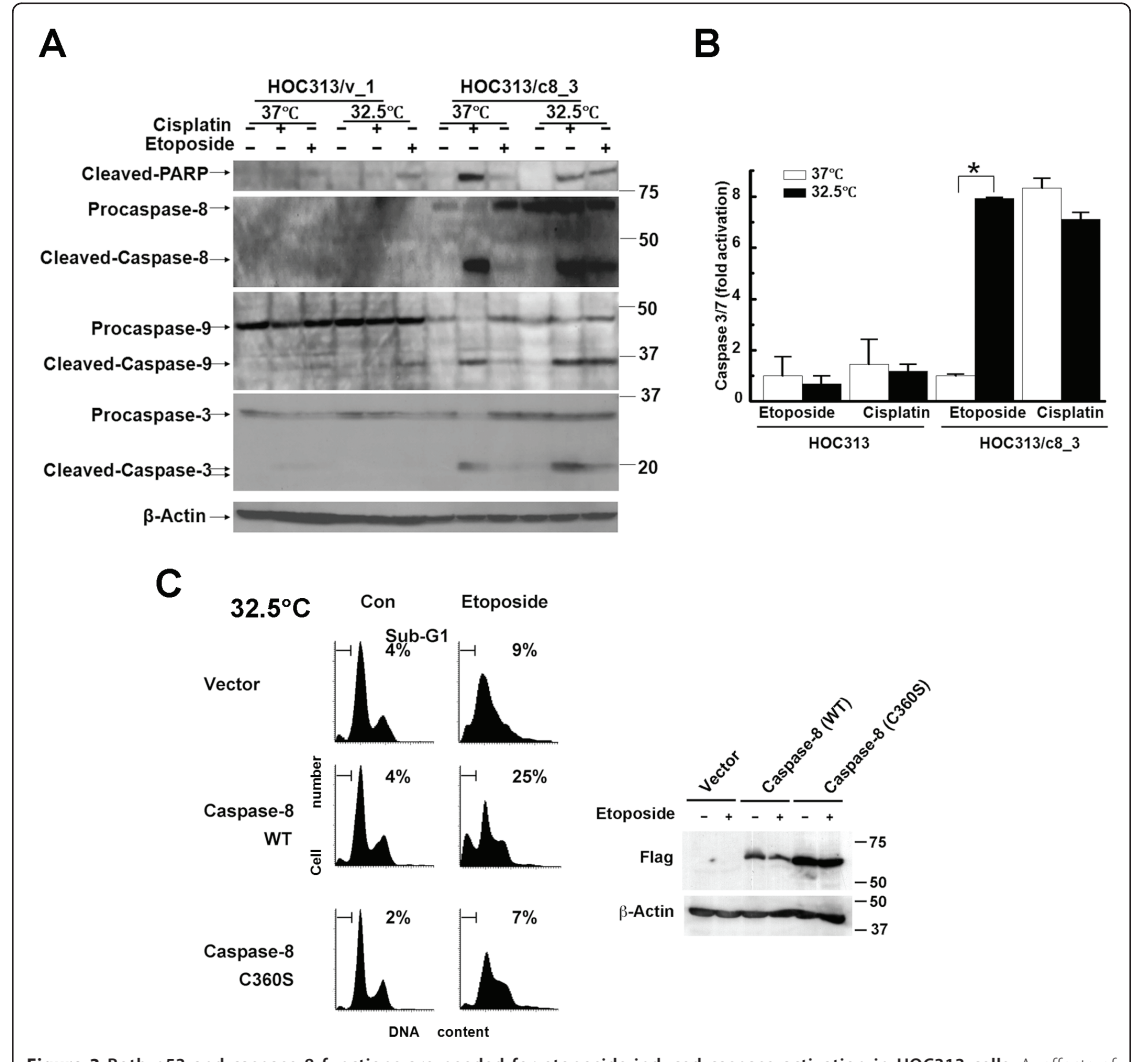
**Both p53 and caspase-8 functions are needed for etoposide-induced caspase activation in HOC313 cells**. A, effects of temperature downshift on the cleavage of the procaspases and PARP in drug-treated HOC313 cells and HOC313/c8_3 cells. The cells treated with drugs as in Figure 2B were subjected to Western blotting for PARP, and the caspases-8, -9 and -3. β-actin was used as a loading control. B, effects of temperature downshift on caspase 3/7 activities in drug-treated HOC313 cells and HOC313/c8_3 cells. The cells treated with drugs as in Figure 2B for 12 h were subjected to Caspase-Glo 3/7 assay. Results are expressed as fold induction compared with untreated control. The bars show the SD. The asterisk indicates a significant difference (p < 0.05) between 32.5°C and 37°C in etoposide-treated HOC313/c8_3 cells (*t*-test). C, the caspase-8 (C360S) mutant did not induced apoptosis in etoposide-treated HOC313 cells at 32.5°C. HOC313 cells were transiently transfected with the indicated expression vectors by electroporation. Twenty-four hours after transfection, cells were treated with etoposide (100 μg/ml) at 32.5°C. DNA profiles were analyzed 40 h after drug treatment as in Figure 2B (left panel). Caspase-8 expression was analyzed by Western blot using anti-FLAG antibody 24 h after drug treatment (right panel).

We examine the roles of p53 and caspase-8 functions in the cleavage of procaspases and PARP, a marker of apoptosis, by Western blotting. Consistent with the results of LSC analysis, p53 restoration by temperature downshift resulted in the cleavage of the transfected Flag-tagged procaspase-8 as well as the cleavage of the procaspases-3, and -9, and PARP following etoposide treatment in caspase-8-reconstituted HOC313/c8_3 cells (Figure 3A). However, no or little cleavage of these procaspases and PARP was observed at 37°C. Furthermore, while p53 restoration by itself induced the proapoptotic gene transcription and Ser46 phosphorylation (Figure 1A and 1B), it induced only a weak cleavage of procaspase-9 and there was no detectable procaspase-3 cleavage in control HOC313/v_1 cells lacking functional caspase-8. By contrast, cisplatin treatment led to cleavage in a manner independent of p53 restoration in HOC313/c8_3 cells. Consistent with these results, p53 restoration resulted in an 8-fold increase of caspase-3/7 activity after etoposide treatment, whereas the activity was increased regardless of p53 function after cisplatin treatment (Figure 3B). These results demonstrate that both caspase-8 and p53 functions are critical for etoposide-induced caspase-9 activation, and also the subsequent cleavage of procaspase-3 and PARP in HOC313 cells. To confirm the requirement of caspase-8 activity in the etoposide-induced apoptosis, HOC313 cells were transiently transfected with the empty vector, wild-type (WT), or C360S inactive mutant of Flag-tagged caspase-8 by electroporation, then treated with etoposide at 32.5°C. Transfection of caspase-8 (WT) induced apoptosis (sub-G_1 _population = 25%), whereas that of the caspase-8 (C360S) mutant did not increase sub-G1 populations compared to the vector transfection (7% vs 9%) (Figure 3C).

### p73 is responsible for etoposide-induced caspase-8 activation and apoptosis in drug-sensitive Ca9-22 cells

**Figure 4:**
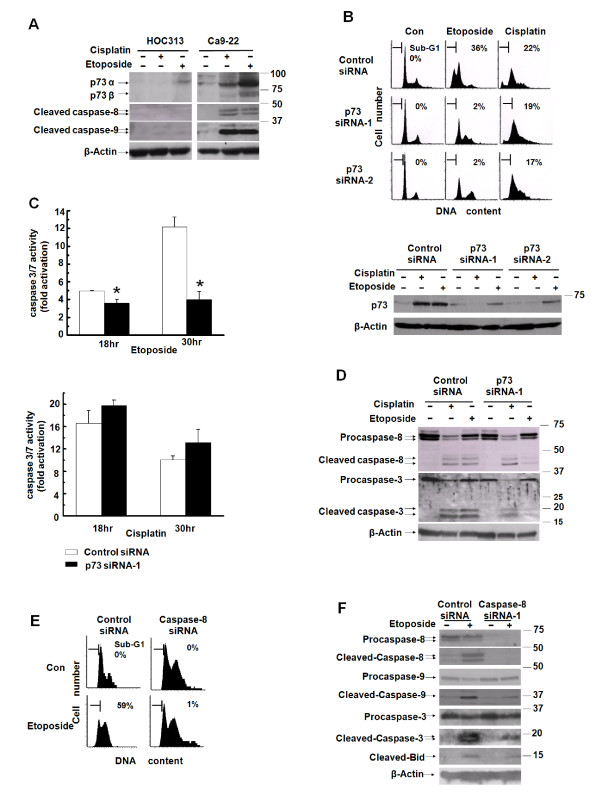
**p73 is responsible for etoposide-induced caspase-8 activation and apoptosis in drug-sensitive Ca9-22 cells**. A, the induction of endogenous p73 following drug treatment. Cells treated with the drugs as in Figure 2B were subjected to Western blotting with p73, caspase-8 or caspase-9 antibodies. B, C and D, Ca9-22 cells were transfected with either p73 or control siRNA. At 48 h after siRNA transfection, cells were treated with cisplatin (5 μg/ml) or etoposide (50 μg/ml). DNA profiles were analyzed by LSC 30 h after drug treatment (B, upper panel). The horizontal and vertical axes represent the DNA content and cell number, respectively. Apoptotic cell population is indicated as a percentage of the sub-G_1 _fraction. p73 expression was analyzed by Western blot 30 h after drug treatment (B, lower panel). Caspase 3/7 activity was analyzed at the indicated time after drug treatment (C). Results are expressed as fold induction compared with untreated control. The bars represent the SD. The asterisk indicates a significant difference (p < 0.05) between p73 siRNA-1 and control siRNA treatment cells (*t*-test). The cleavage of procaspases was analyzed by western blot with caspases-3 and -8 antibodies 24 h after drug treatment (D). E and F, Ca9-22 cells were transfected with either caspase-8 or control siRNA. At 48 h after siRNA transfection, cells were treated with etoposide (100 μg/ml). DNA profiles were analyzed by LSC 24 h after drug treatment as in B (E). The expression and cleavage of procaspases and Bid was analyzed by western blot with caspases-3, -8, -9, and Bid antibodies 24 h after drug treatment (F). β-actin was used as a loading control.

It has been shown that the p53 homologue p73 is induced by genotoxic drugs, and plays a critical role in p53-independent apoptosis in p53-deficient tumors [37,38]. Etoposide treatment up-regulated the p53-target genes in Ca9-22, but not in HOC313 cells under non-permissive conditions (37°C) (Figure 1A). Therefore, we examined the expression levels of the p73 protein following drug treatment in HOC313 and Ca9-22 cells. Western blot analysis revealed that etoposide induced the expression of p73α and p73β much more potently than did cisplatin, accompanied by the cleavage of procaspases-8 and -9 in Ca9-22 cells (Figure 4A). However, induction of p73 was substantially reduced in HOC313 cells relative to Ca9-22 cells after treatment with either etoposide or cisplatin.

The fact that p53R248W mutation in Ca9-22 cells is a hotspot mutation resulting in loss of sequence-specific DNA binding and apoptotic functions suggests that p73 plays a critical role in etoposide-induced apoptosis in Ca9-22 cells. To test this possibility, we examined the effects of p73 knockdown on etoposide-induced apoptosis in drug-sensitive Ca9-22 cells by using two p73 small interfering RNAs (siRNAs) that target different p73 sequences (p73 siRNA-1 and -2). LSC analysis revealed that siRNA-based p73 knockdown (Figure 4B, lower panel) decreased the sub-G_1 _population in etoposide-treated Ca9-22 cells, but had more subtle effects on cisplatin-treated cells (Figure 4B, upper panel). The caspase 3/7 assays performed 18 hr and 30 hr after drug treatment consistently indicated that p73 siRNA blocked caspase 3/7 activity in etoposide- but not cisplatin-treated Ca9-22 cells (Figure 4C). Consistent with this, western blotting verified that p73 siRNA blocked the cleavage of procaspases-3, and -8 after etoposide, but had no or very slight effects on the cleavage of these procaspases after cisplatin treatment (Figure 4D). Furthermore, caspase-8 knockdown by siRNA blocked etoposide-induced apoptosis (Figure 4E) and also the cleavage of procaspase-9 and -3 in Ca9-22 cells (Figure 4F), confirming that caspase-8 activity is required for etoposide-induced apoptosis in Ca9-22 cells. Collectively, these results indicate that p73 is responsible for etoposide- but not cisplatin-induced caspase-8 activation and apoptosis in p53-deficient Ca9-22 cells, and suggest that reduction of p73 expression, at least in part, contributes to etoposide resistance in HOC313 cells.

### Caspase-9 is functionally relevant in caspase-8 activation and the induction of apoptosis by etoposide

**Figure 5:**
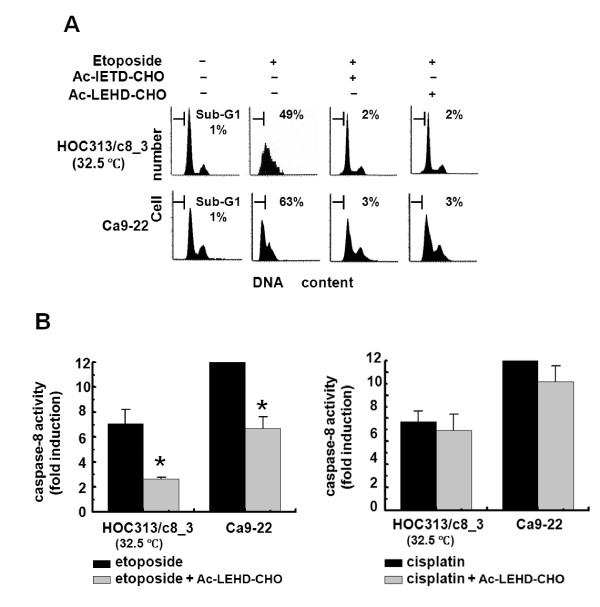
**Caspase-9 is functionally relevant in etoposide-induced caspase-8 activation and apoptosis**. A and B, effects of caspase inhibitors on caspase-8 activation and the induction of apoptosis by etoposide in HNSCC cell lines. HOC313/c8_3 cells and Ca9-22 cells were incubated at 32.5°C and 37°C respectively with the indicated caspase inhibitors (80 μM) for 1.5 h, followed by treatment with cisplatin (10 μg/ml) or etoposide (100 μg/ml) for 24 h. The DNA profiles were analyzed by LSC as in Figure 2B (A). Caspase-8 activity was analyzed by Caspase-Glo 8 assay (B). Results are expressed as fold induction compared with untreated control. The bars represent the SD. The asterisk indicates a significant difference (p < 0.05) between the presence and absence of Ac-LEHD-CHO in etoposide-treated cells (*t*-test).

The results presented above indicate that p53/p73 functions are required for etoposide-induced caspase-8 activation and apoptosis in the HNSCC cell lines. Caspase activation by p53/p73 occurs through the activation of the mitochondrial pathway, leading to the activation of caspase-9. Furthermore, caspase-8 can be activated downstream of caspase-9 by the activated caspases-3 and -6. To explore the mechanisms of p53/p73-dependent caspase-8 activation and the apoptosis induced by etoposide, we examined the functional role of caspase-9 in these events by using Ac-IETD-CHO or Ac-LEHD-CHO, two peptide inhibitors specific for caspases-8 and -9, respectively. Consistent with the results of caspase-8 knockdown (Figure 4E), LSC analysis confirmed that the caspase-8 inhibitor blocked the apoptosis induced by etoposide in Ca9-22 and p53-restored (32.5°C) HOC313/c8_3 cells (Figure 5A). Importantly, the caspases-9 inhibitor also blocked etoposide-induced apoptosis in both cell lines. Furthermore, enzymatic measurement of caspase-8 activities revealed that the caspase-9 inhibitor blocked etoposide-induced caspase-8 activation in both cell lines, indicating the requirement of caspase-9 function for caspase-8 activation induced by etoposide (Figure 5B). However, the caspases-9 inhibitor had only a slight effect on caspase-8 activation by cisplatin, which is consistent with our previous observations that cisplatin activates caspase-8 via the death receptor pathway in these cell lines [42].

**Figure 6:**
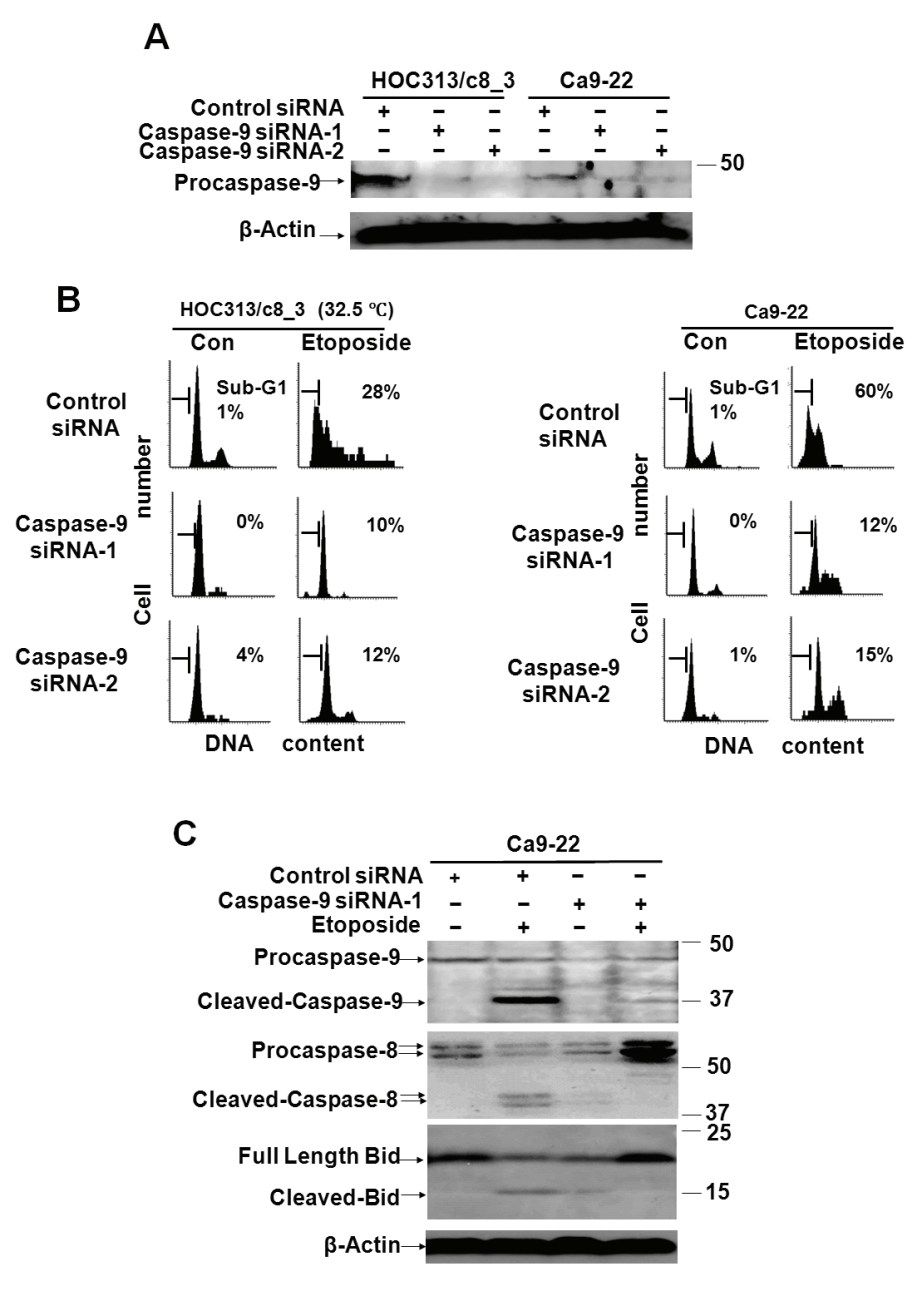
**Caspase-9 is functionally relevant in etoposide-induced caspase-8 activation and apoptosis**. A and B, cells were transfected with either caspase-9 or control siRNA. At 48 h after siRNA transfection, cells were treated with cisplatin (10 μg/ml) or etoposide (100 μg/ml). Caspase-9 expression was analyzed by Western blot 24 h after drug treatment (A). The DNA profiles were analyzed by LSC 24 h after drug treatment (B). The horizontal and vertical axes represent the DNA content and cell number, respectively. Apoptotic cell population is indicated as a percentage of the sub-G_1 _fraction. The cleavage of procaspase-9 and Bid was analyzed by Western blot with caspases-9 and Bid antibodies 24 h after drug treatment (C). β-actin was used as a loading control.

The requirement of caspase-9 in etoposide-induced caspase-8 activation and apoptosis was further confirmed by transfection with two caspase-9 siRNAs that target two different caspase-9 sequences (caspase-9 siRNA-1 and -2) into Ca9-22 and HOC313/c8_3 cells. LSC analysis revealed that caspase-9 siRNA (Figure 6A) decreased the sub-G_1 _populations in the Ca9-22 and p53-restored HOC313/c8_3 cells treated with etoposide (Figure 6B). This was paralleled by the inhibition of caspase-8 cleavage by caspase-9 siRNA, as demonstrated by Western blotting (Figure 6C). Collectively, these results demonstrate that p53/p73-dependent caspase-8 activation and apoptosis induction by etoposide are mediated through the activation of the mitochondrial pathway in these HNSCC cell lines.

### Caspase-8 reconstitution activates the p53-mediated mitochondrial pathway induced by etoposide

**Figure 7:**
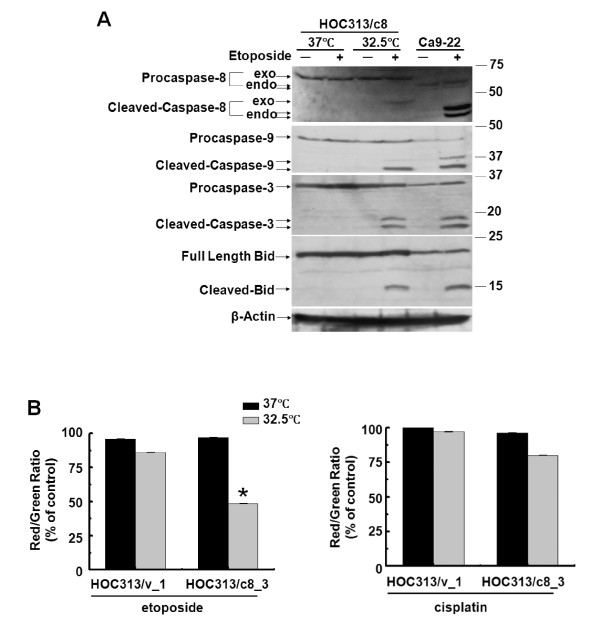
**Caspase-8 reconstitution activates the p53-mediated mitochondrial pathway induced by etoposide**. A, cleavage of procaspase-9 and Bid were analyzed by Western blot with caspase-9 and Bid antibodies 24 h after drug treatment. Exo and endo represent exogenous and endogenous, respectively. B, effects of drug treatment on the mitochondrial membrane potential (ΔΨm) in HOC313/v_1 and HOC313/c8_3 cells at 32.5°C or 37°C. Cells treated with cisplatin (10 μg/ml) or etoposide (100 μg/ml) for 10 h were subjected to JC-1 assay as described in Materials and methods. To measure ΔΨm, the ratio of red to green fluorescence of JC-1 was calculated for each sample and compared with the untreated control JC-1 fluorescence ratio at each temperature. Results are the means ± SD from three independent experiments. The bars represent the SD. The asterisk indicates a significant difference (p < 0.05) between 32.5°C and 37°C in etoposide-treated HOC313/c8_3 cells (*t*-test).

It has been reported that activation of caspase-8 initiates a positive feedback loop that amplifies the mitochondrial pathway via cleavage of proapoptotic Bid, leading to depolarization of the mitochondria and subsequent caspase-9 activation. As shown in Figure 7A, etoposide treatment induced the cleavage of Bid into tBid in Ca9-22 cells, and etoposide treatment, together with p53 restoration at 32.5°C, led to Bid cleavage in caspase-8 reconstituted HOC313/c8_3 cells in parallel with the cleavage of the procaspases-3, and -8, -9. Furthermore, caspase-9 siRNA suppressed etoposide-induced cleavage of Bid in Ca-9-22 cells (Figure 6C). Finally, we examined the alteration of the mitochondrial membrane potential (ΔΨm) of HOC313/v_1 and HOC313/c8_3 cells following drug treatment by JC-1 assay. Figure 7B shows that etoposide treatment and p53-restoration at 32.5°C led to the reduction of ΔΨm (48%) in caspase-8-reconstituted HOC313/c8_3, but not in HOC313/v_1 cells, whereas cisplatin treatment had a little effect on the ΔΨm at either temperature. Taken together, these results indicate that caspases-8 plays a critical role in etoposide-induced p53-dependent depolarization of the mitochondria.

## Discussion

While caspase-8 and the p53 family members play critical roles in triggering apoptosis induced by genotoxic drugs, the functional relevance of caspase-8 in p53/p73-dependent apoptosis has not been fully understood. In the present study, we investigated the relationship of caspase-8 and the p53 status with drug-induced apoptosis in HNSCC cells. A capase-8 deficient HOC313 cell line carrying temperature-sensitive p53G285K mutant was a useful tool to address this issue. Here, we provide evidence that caspase-8 is an essential mediator of the p53/p73-dependent apoptosis induced by etoposide in HNSCC cells.

The different lines of experiments clearly demonstrate the functional relevance of caspase-8 in p53/p73-dependent apoptosis induced by etoposide in HNSCC cells. First, the restoration of p53 function, by itself, induced proapoptotic PUMA and Noxa, but did not activate casapases-3 and -9 or induce apoptosis in HOC313 cells following etoposide treatment. However, in addition to p53 restoration, caspase-8 reconstitution was required for sensitizing etoposide-induced apoptosis, mitochondrial depolarization, and the cleavage of procaspases-3, -9, and Bid in HOC313 cells. Second, in drug-sensitive Ca9-22 cells, p73 siRNA blocked etoposide-induced apoptosis, caspase-8 cleavage, and caspase-3/7 activation, which correlated with the observed reduction of p73 expression in etoposide-resistant HOC313 cells. Finally, both the caspase-8 inhibitor Ac-IETD-CHO and caspase-8 siRNA inhibited etoposide-induced apoptosis in Ca9-22 cells. The consistency on the results obtained from both the etoposide-resistant HOC313 and etoposide-sensitive Ca9-22 HNSCC cell lines suggests that our results may reflect characteristics of HNSCC cells, rather than a cell-type-specific phenomenon that occurs only in HOC313 cells. Although drug-induced caspase-8 activation in death receptor-independent apoptosis has been reported in several types of cells, our data demonstrate that p53 and p73 act as key upstream regulators of caspase-8 in etoposide-induced apoptosis in HNSCC cells. In agreement with this, etoposide treatment led to up-regulation of the proapoptotic p53-target genes PUMA and Noxa in both Ca9-22 and p53-restored HOC313 cells. Furthermore, neither death ligand nor death receptor mRNA was induced by etoposide in HOC313 and Ca9-22 cells [42]. While the available information on the involvement of caspase-8 function in p53- and/or p73-dependent apoptosis is limited, caspase-8-mediated cleavage of Bid has been observed during p53-dependent apoptosis induced by neutron [46]. In addition, p53 has been shown to transactivate the caspase-8 promoter [47], and p53-mediated up-regulation of caspase-8 and sensitization toward death receptor-mediated apoptosis was observed in certain tumor cell lines with down-regulated caspase-8 as well as TRAIL-resistant primary tumor cells following drug treatment [48]. However, up-regulation of caspase-8 mRNA was not detected in p53-deficient Ca9-22 cells following etoposide treatment (data not shown).

**Figure 8:**
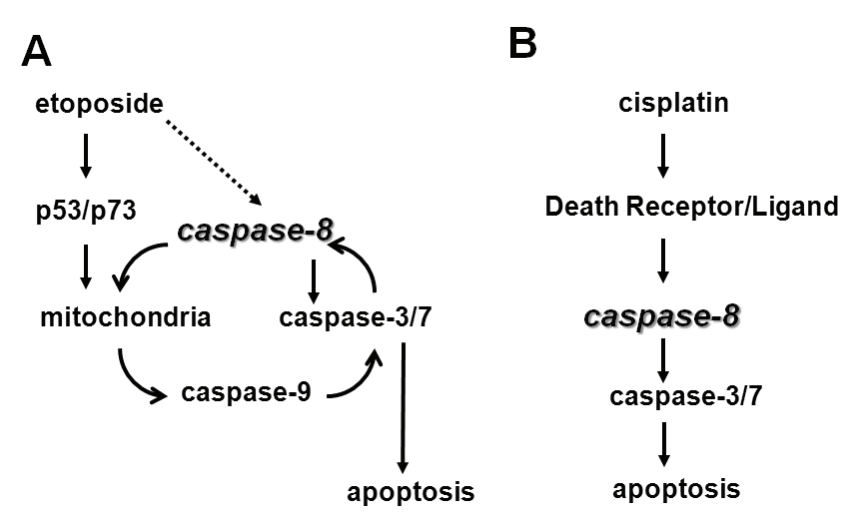
**Model of the drug-specific activation of apoptotic pathways in HNSCC cells**. Caspase-8 plays critical roles in inducing both death receptor- and mitochondria-mediated apoptosis in HNSCC cells. The caspase-8-mediated signal amplification of the mitochondrial pathway is important for determining the sensitivity to etoposide-induced and p53/p73-mediated apoptosis (A). Caspase-8 also mediates cisplatin-induced and death receptor-mediated apoptosis (B) [42].

We show that p53/p73-dependent caspase-8 activation is mediated by caspase-9 in etoposide-induced apoptosis in HNSCC cells. Our results clearly demonstrate that the caspase-9 inhibitor Ac-LEHD-CHO, or caspase-9 siRNA, blocked etoposide-induced apoptosis in both HOC313/c8_3 and Ca9-22 cell lines. Consistent with this, Ac-LEHD-CHO blocked caspase-8 activation, and caspase-9 siRNA also blocked the cleavage of procaspase-8 and Bid, indicating that caspase-8 activation lies downstream of the mitochondrial pathway, as reported in previous studies [14-19]. In turn, caspase-8 activation was required for p53/p73-dependent Bid cleavage, full activation of caspase-9, and mitochondrial depolarization. In contrast, p53 restoration, by itself, induced little if any mitochondrial depolarization, and no or weak cleavage of procaspases-3 and -9 in caspase-8-deficient HOC313/v_1 cells. In addition, there was no difference in the expression level of Inhibitor of apoptosis proteins (IAPs), such as Survivin, XIAP, and c-IAP, between etoposide-sensitive Ca9-22 and -resistant HOC313 cells (data not shown), indicating that caspase-8 loss does not lead to the accumulation of IAPs in HOC313 cells. Furthermore, the apoptotic inhibitor cellular FLICE-inhibitory protein (c-FLIP) was not detected in etoposide-resistant HOC313 cells in both permissive and non-permissive temperatures (data not shown). Thus, results presented in this study are consistent with a model in which the initial activation of caspase-9 is amplified via caspase-8-mediated positive feedback loop, which is required for the full activation of the caspases in p53/p73-dependent apoptosis induced by etoposide in HNSCC cells (Figure 8A). If this is the case, a dominant negative mutant of FADD may not be able to rescue etoposide-induced apoptosis in HNSCC cells. Alternatively, it remains a possibility that caspase-8 might be weakly activated by etoposide through the death receptor pathway in HNSCC cells, and works together with p53/p73 in the caspase-8-mediated positive feedback loop. Our results indicate that, in the absence of p53/p73 functions, the amounts of cleaved caspase-8 and -9 are little, and thus caspase-8-mediated positive feedback loop can not work in HNSCC cells.

Our results are also consistent with previous studies indicating that p73 is a critical determinant of drug-induced cell death in HNSCC [38,39]. We show that p73 siRNA blocked etoposide-induced apoptosis, procaspase-8 cleavage, and caspase-3/7 activation in Ca9-22 cells. Moreover, in line with a previous study demonstrating that p73 induces mitochondria-mediated apoptosis through PUMA transactivation [49], etoposide induced p73 protein and *Noxa *and *PUMA *mRNA in Ca9-22 cells. These results indicate that, in the absence of functional p53, p73 plays a key role in etoposide-induced caspase-8 activation and apoptosis in Ca9-22 cells. In this regard, the reduction of p73 expression and lack of *Noxa *and *PUMA *mRNA induction in etoposide-treated HOC313 cells under non-permissive conditions of p53G285K suggest that this attenuated p73 induction contributes to etoposide resistance in HOC313 cells. In agreement with this, the loss of p73 induction by genotoxic drugs has also been reported in chemoresistant bladder and ovarian cancer cells [50,51]. Although the mechanism underlying the attenuated induction of p73 in HOC313 cells is unclear, methylation-associated gene silencing has been demonstrated in the p73 promoter in leukemia cells [52]. Additionally, while p63 has been shown to influence chemosensitivity in HNSCC cells [40], the expression of neither the TA nor ΔN p63 isoform was detected in HOC313 cells; in Ca9-22 cells, while the protein levels of these isoforms were detectable, but they did not change following drug treatment (data not shown).

The apoptosis provoked by genotoxic drugs requires the death-receptor and mitochondrial apoptotic pathways, but which of the pathways is critical for the response to a particular drug are not fully understood. Although the precise signalling mechanisms underlying the response to etoposide and cisplatin remains unclear at present, we show that the signals upstream of caspase-8 activation induced by cisplatin are different from those by etoposide in HNSCC cells. The results presented here, together with our previous study, demonstrate that p53/p73 function is dispensable for the caspase-8 activation and apoptosis induction by cisplatin. Furthermore, cisplatin was less effective for the up-regulation of proapoptotic p53-target genes and mitochondrial depolarization than etoposide. Previous studies have demonstrated that caspase-8 activation by genotoxic drugs is mediated by death receptor signaling [5-10]. In line with this, we have reported that cisplatin, but not etoposide induced death ligand mRNA expression, such as TNF-α and TRAIL, in Ca9-22 and HOC313 cells [42]. Furthermore, it has been reported that the induction of TNF-α by NF-κB plays a critical role in cisplatin-induced apoptosis in HNSCC cells [53]. Thus, our results support the view that cisplatin triggers apoptosis in a death receptor-dependent manner in HNSCC cells (Figure 8B). Nevertheless, the cleavage of procaspase-9 was observed in cisplatin-treated HOC313/c8_3 cells, indicating the activation of the mitochondrial pathway during cisplatin-induced apoptosis. However, the caspase-9 inhibitor Ac-LEHD-CHO had only a slight effect on cisplatin-induced caspase-8 activation (Figure 5B, right panel).

## Conclusions

The results presented here clearly demonstrate that p53 and p73 can act as upstream regulators of caspase-8, and that caspase-8 plays a critical role for the execution of the p53/p73-dependent apoptosis induced by etoposide in HNSCC cells. Our data suggest that caspase-8-mediated feedback signal amplification is required for full activation of the caspases in the p53/p73-dependent apoptosis induced by etoposide in HNSCC cells. Although caspase-8 mutation is rare in HNSCC, the understanding of the molecular mechanisms underlying chemoresistance in tumor cells may help to design new strategies for chemotherapy. In this regard, these results provide new insight into the molecular cascade of apoptosis signaling in HNSCC cells.

## Methods

### Cell culture

The human HNSCC cell lines HOC313 (p53G285K; caspase-8R68X) [42] and Ca9-22 (p53R248W; caspase-8 wild type) were established at the Second Department of Oral and Maxillofacial Surgery, Faculty of Dentistry, Tokyo Medical and Dental University, and maintained in our laboratory as described [54,55]. HOC313 derivative HOC313/c8_3 and control HOC313/v_1 cell lines have been described previously [42]. All HNSCC cell lines and the Saos-2 osteosarcoma cell line were maintained in Dulbecco's Modified Eagle's Medium (Nissui Pharmaceutical, Tokyo, Japan) supplemented with 10% fetal bovine serum and gentamicin at 37°C (unless 32.5°C is specifically indicated) in the presence of 5% CO2.

### CAT assay

Saos-2 cells were transfected with 1 μg of reporter plasmid p53CONTK-CAT, together with 1 μg of either pCMV-p53 or an empty vector. Twelve hours after transfection, the cells were left at 37°C or shifted to 32.5°C. After continued incubation for 24 h, the cells were subjected to CAT assay as previously described [37]. The amounts of acetylated [14C] chloramphenicol were quantitated using the Phosphor Autoradiography System BAS2000 (Fuji Film, Tokyo, Japan).

### Chemicals

Cisplatin (Wako, Osaka, Japan) and etoposide (Sigma-Aldrich, St. Louis, MO) were dissolved in DMSO just before use in each experiment. The caspase-9 inhibitor Ac-LEHD-CHO and caspase-8 inhibitor Ac-IETD-CHO were purchased from Peptide Institute (Osaka, Japan).

### Western blotting

Western blotting analysis was performed as previously described [38], using the following antibodies at dilutions of 1: 500 to 1:1000: anti-p53 (sc-126), anti-p53-phospho-ser15(# 9284), anti-p53-phospho-ser46 (#2521) (Cell signaling technology, Beverly, MA), anti-p21 (sc-469, Santa Cruz Biotechnology, Santa Cruz, CA), anti-beta actin (clone AC-15, Sigma-Aldrich), anti-caspase-8 (clone 5F7, MBL, Nagoya, Japan), anti-PARP (#9546), anti-caspase-9 (#9502), anti-Bid (#2002) (Cell signaling technology, Beverly, MA), anti-caspase-3 (sc-7148), and anti-p73 antibodies (Ab-4, Neomarkers, Fremont, CA).

### Real-time reverse transcriptase PCR measurement of RNA

Total RNA was isolated by using TRI reagent (sigma) according to the manufacturer's instructions. RT was performed using 5 μg of total RNA using random primers and MMLV reverse transcriptase (TOYOBO, Osaka, Japan). Quantitative real-time RT-PCR analysis was performed on the LightCycler 480 instrument (Roche Applied Science, Mannheim, Germany) in triplicate using TaqMan Gene Expression Assays for PUMA, Noxa, p21/CDKN1A, and 18s rRNA (Hs00248075_m1, Hs00560402_m1, Hs00355782_m1, and Hs03003631_g1, respectively) (Applied Biosystems, Foster City, CA, USA) and the LightCycler 480 Probes Master kit (Roche Applied Science), according to the manufacturer's instructions. The relative expression of mRNA, normalized to 18srRNA, was calculated using the 2 ^(-ΔΔCP) ^method.

### WST-1 and caspase 3/7 and caspase-8 assays

Cells treated with increasing doses of anticancer drugs were subjected to WST-1 assay as previously described [38]. Cell viability was determined as a percentage of the control. Data represent the average of six independent experiments, each performed in triplicate. Caspase 3/7 and caspase-8 activity were determined using Caspase-Glo 3/7 and Caspase-Glo 8 assay kits (Promega, Madison, WI) according to the manufacturer's instructions. The data represent the average of three independent experiments.

### Cell cycle analysis

Drug-treated cells were fixed with 75% ethanol, and then stained for DNA content as described previously [39]. Cell cycle profiles were analyzed using a laser scanning cytometer (LSC101; Olympus, Tokyo, Japan).

### Plasmid

The expression vector for 3XFLAG caspase-8 WT (pCMV-FLAG-caspase-8) has been described previously [42]. The C360S mutation was introduced into pCMV-FLAG-caspase-8 with appropriate mutagenesis primers. The construct was confirmed by DNA sequencing.

### Transient Transfection

HOC313 cells (3 × 10^6^) were transfected with 6 μg of each DNA construct using a pipette-type electroporator (MicroPorator MP-100, Digital Bio) (pulse voltage; 1600 V, pulse width; 20 ms, pulse number; 1), and plated into two 6 cm dishes. Twenty-four hours after transfection, cells were treated with etoposide (100 μg/ml), then harvested at the indicated time.

### Measurement of mitochondrial membrane potential

The JC-1 mitochondrial membrane potential assay kit (Cayman Chemical Co. Ann Arbor, MI) was used to measure ΔΨm according to manufacturer's instructions. Cells were treated with drugs in 12-well plates for 10 hours, and then incubated with 0.1 volume of JC-1 Staining Solution to growth media for 15 min. Thereafter, the cells were washed twice with supplied assay buffer, and the plates were immediately read using a multilabel counter (1420 ARVO MX, PerkinElmer, Tokyo, Japan) with the excitation (Ex) and emission (Em) wavelengths set at 530 nm Ex/590 nm Em for red J-aggregate fluorescence, and 485 nm Ex/530 nm Em for green fluorescence. The red/green fluorescence ratios were calculated after the fluorescence values had been corrected for the background.

### siRNA transfection

Stealth siRNA duplexes targeting p73 (p73 siRNA-1: 5'-UCUGCUUGAAGGCACGCUUGCUGGC-3' and p73 siRNA-2: 5'-AGUACGUGUCCUCGUCUCCAUGCCG-3'), Caspase-8 (Caspase-8 siRNA: 5'-AUAACAUCAAGGCAUCCUUGAUGGG-3'), and Caspase-9 (Caspase-9 siRNA-1: 5'-AUGAUCAGCUGCCUGGCCUGAUCCC-3' and Caspase-9 siRNA-2: 5'-UUUGCUGCUUGCCUGUUAGUUCGCA-3'), and negative control duplexes (12935-400) were purchased from Invitrogen. Cells were reverse transfected with siRNA at approximately 30-50% confluency using Lipofectamine RNAiMAX (Invitrogen) according to the manufacturer's recommendations, and incubated for 48 hr prior to drug treatment.

### Statistical analysis

The data are expressed as the mean ± SD. Significance was calculated using Student's *t*-test. Values of *P *< 0.05 were considered to be significant.

## List of abbreviations

HNSCC: head and neck squamous carcinoma cells; TRAIL: TNF-related apoptosis-inducing ligand; NF-κB: nuclear factor κ-B; MMLV: Moloney murine leukemia virus; CAT: chloramphenicol acetyltransferase; tBid: truncated Bid; ΔΨm: mitochondrial membrance potential; PARP: poly (ADP-ribose) polymerase; IAP: inhibitor of apoptosis protein.

## Competing interests

The authors declare that they have no competing interests.

## Authors' contributions

JL and MAI designed the experiments. JL carried out the majority of experiments, performed the relevant data analysis, and wrote the drafts of the manuscript. HU provided valuable reagents and advice. NT carried out the cell viability and CAT assays and contributed to the critical revision of the manuscript. MAI supervised the experiments and finalized the manuscript. All authors read and approved the final manuscript.
